# Examination of the ocean as a source for atmospheric microplastics

**DOI:** 10.1371/journal.pone.0232746

**Published:** 2020-05-12

**Authors:** Steve Allen, Deonie Allen, Kerry Moss, Gaël Le Roux, Vernon R. Phoenix, Jeroen E. Sonke

**Affiliations:** 1 Centre for Water, Environment, Sustainability and Public Health, Department of Civil and Environmental Engineering, University of Strathclyde, Glasgow, United Kingdom; 2 EcoLab (Laboratoire Ecologie Fonctionnelle et Environnement), ENSAT, UMR-CNRS 5245, Castanet Tolosan, Toulouse, France; 3 Ocean Sciences, Nelson Mandela University, Port Elizabeth, South Africa; 4 Geosciences Environnement Toulouse, CNRS/OMP/Université de Toulouse, Toulouse, France; VIT University, INDIA

## Abstract

Global plastic litter pollution has been increasing alongside demand since plastic products gained commercial popularity in the 1930’s. Current plastic pollutant research has generally assumed that once plastics enter the ocean they are there to stay, retained permanently within the ocean currents, biota or sediment until eventual deposition on the sea floor or become washed up onto the beach. In contrast to this, we suggest it appears that some plastic particles could be leaving the sea and entering the atmosphere along with sea salt, bacteria, virus’ and algae. This occurs via the process of bubble burst ejection and wave action, for example from strong wind or sea state turbulence. In this manuscript we review evidence from the existing literature which is relevant to this theory and follow this with a pilot study which analyses microplastics (MP) in sea spray. Here we show first evidence of MP particles, analysed by μRaman, in marine boundary layer air samples on the French Atlantic coast during both onshore (average of 2.9MP/m^3^) and offshore (average of 9.6MP/m^3^) winds. Notably, during sampling, the convergence of sea breeze meant our samples were dominated by sea spray, increasing our capacity to sample MPs if they were released from the sea. Our results indicate a potential for MPs to be released from the marine environment into the atmosphere by sea-spray giving a globally extrapolated figure of 136000 ton/yr blowing on shore.

## Missing plastic in the marine microplastic models

Since the first evidence of anthropogenic plastic litter affecting sea birds in the 1960’s [1,2] there has been a steadily growing awareness that plastics are becoming a major pollutant. According to the plastic industries figures, around 359 million tons of plastic was manufactured globally in 2018 (up from 334 million tons in 2016) [3], of which 60 million tons were produced in Europe. Mattsson et al. (2015) estimate that around 10% of all plastic produced is lost to the sea each year [4]. A 2010 estimation suggests between 4.8–12.7 million tons of plastic entered the oceans from coastal and terrestrial areas, with up to 92% of this being <4.75mm in size [5].

That this figure is now 10 years old and as around half of all plastics produced has been in the last 15 years, it is likely that figure would significantly underestimate current levels [6,7]. A 2015 estimate by van Sebille et al. suggests 93000–236 000 tons of plastic floating in the world’s surface oceans [8]. Koelmans et al. (2017) [9] simulations suggest 99.8% of oceanic plastic has sunk below the ocean surface layer (OSL), however this figure is primarily based on the plastic not being visible or identifiable in surface samples. Various oceanic plastic transport models created, such as Maximenko et al.(2012) [10], van Sebille et al. (2015) [8]_,_ Jambeck et al. (2015) [11] and Wichmann et al. (2018) [12], make mention of “leaky basins” to explain areas that do not contain the plastic concentrations or quantities the models predict. In spite of the great effort to model oceanic plastic transport and sinks, there does not seem to be a definitive answer for the missing plastics and considering the potential for atmospheric plastic to be depositing in the ocean, the missing plastic quantity could be much greater.

The ocean or marine environment is generally been considered a microplastic (MP) sink, with cities, human activities (including waste mismanagement) and industry being primary MP pollution sources. Early transport studies have identified MP moving from cities to rivers, rivers to sea and most recently atmospheric transport of MP across terrestrial environments and out to sea [13–16]. With the emergence of atmospheric MP monitoring comes the acknowledgement of local to long-distance atmospheric transport and the potential for MP to reach even remote locations (marine and terrestrial). However, to date there has been no consideration of the oceans as an atmospheric MP source. This research seeks to determine if MP could be leaving the ocean through marine boundary layer interaction with the ocean surface layer. This unexplored secondary source of MP (the sea) and transport pathway (ocean to atmosphere exchange) could help identify at least some of the missing plastic pollution identified in the global marine models. It could also help explain, in part, the occurrence of MP in the air sampled extensive distances offshore (marine air samples [15,16]. While anthropogenic terrestrial atmospheric MP sources are known, and inroads to quantifying their MP emissions are being made, this analysis and pilot study aims to identify if the ocean is a marine MP emission source and take the first steps towards quantifying the influence of this on terrestrial airmass MP. In addition, we discuss the potential impact of MP emissions.

## Ocean to atmosphere particle transfer processes

**Fig 1:**
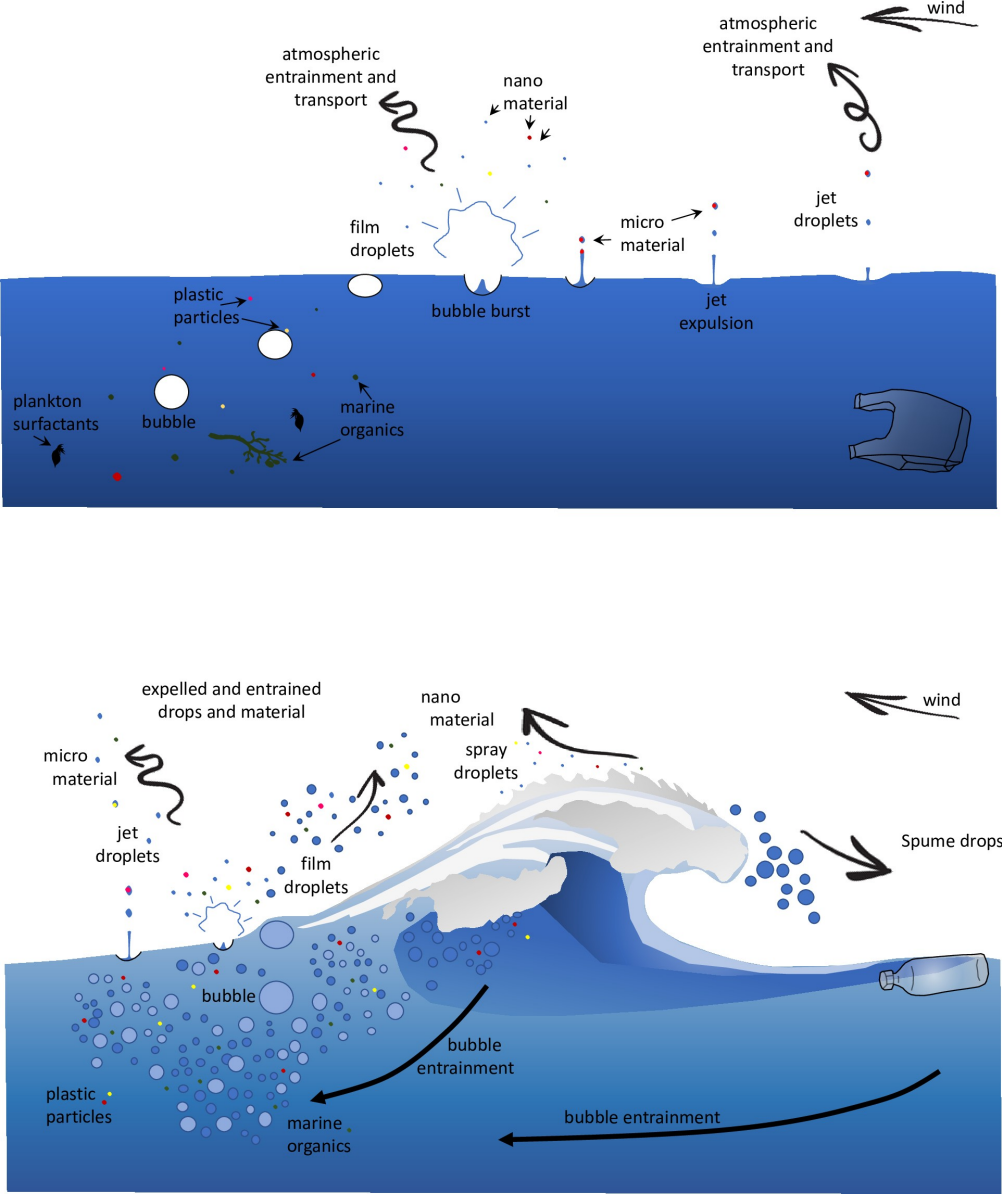
The established SSA and organic matter ocean to atmosphere bubble burst (Fig 1A) and wave exchange (Fig 1B), and the potential micro and nano plastic exchange process mimicking these processes.

There is evidence that MP is making its way to some of the most remote corners of the globe though limited discussion has been made on how it got there. Given the current state of this knowledge we propose a hypothesis on a previously unexamined potential transport vector to advance the discussion. Most marine particles (non-MP) are sea salt (SS) and organic material. Every year approximately 6700-7400Tg of sea salt aerosols (SSA) and organic matter from a few nanometres up to ~20 μm are produced by wave/wind/interaction which are then transported into the atmosphere through convective updrafts [17,18]. Under normal conditions micro and nano size salt particles are ejected from the sea when breaking waves cause bubbles of trapped air to rise to the surface and burst [19] (Fig 1A). The bursting of the unsupported surface of the bubble leaves nano sized particles expelled and suspended in the air available for wind transport [20,21]. With the surface bubble removed the water seeks to fill the void left by the bubble, collision of water from all sides causes the secondary ejection known as a jet to eject the larger micro sized particles [22]. This phenomenon also ejects organic matter which is an important element in cloud formation and precipitation rates (particularly in warm air) [23,24]. Bacteria and virus cells have been well documented traveling in wind and as aerosols across continents and oceans [25] and these organisms have been reported to exit the sea in a similar fashion to SSA [26].

There is a potential for upwelling, through Ekman’s transport, Langmuir spirals and surface gravitational waves drawing water from as deep a 200m below the surface [27], to act as a form of MP supply to the surface and bubble burst ejection process. Most marine particles (non-MP) are sea salt (SS) and organic material. SS are ejected droplets/aerosols and are infinitely available whereas organics are produced in the OSL and are not directly dependent on upwelling supply. MP in contrast can often be found in greater quantities below the surface [28] and the mixing of the surface and deeper marine waters, and associated upwelling processes, may be influential in the availability of MP [29].

## Atmospheric microplastic

To date there have been very few atmospheric MP studies. Of the studies undertaken, presented in recently published reviews [30–32], it is noted that MP particle of fibre, film, foam and fragment shapes have been found in atmospheric deposition and sampled air masses. MP sizes range down to 10μm (lower limit of detection published to date) and up to 5mm but with a greater proportion of sampled particles <500μm [30–32]. Atmospheric MP has been sampled by deposition and active air mass (pumped) sampling, presenting up to 1008 MP/m^2^/day deposition [33] and 60 MP/m^3^ in air mass sampling [34].

Several studies have considered city MP deposition, identifying types and quantities of atmospheric MP deposition in locations including Paris, Dongguan, Shanghai and Hamburg [14,34,35]. Only a few studies have considered remote location MP deposition and a precursor to MP long-distance transport analysis; Tibetan plateau, Arctic snow, Pyrenean Mountains and west Pacific Ocean [16,30,36,37]. Only the study of Pyrenees Mountain MP deposition considered and attempted to model atmospheric transport [36] based on MP deposition findings in the field.

Dris et al. (2016) describe fibres as large as 600μm and smaller than 50 μm collected from atmospheric fallout in the mega-city Paris, France. Their study describes collecting fibres sub 50μm however they did not have the ability to accurately identify the plastics below this level, thus they were excluded from their overall analysis. The source of this fallout was not examined.

The study by Cai et al. (2017) at three locations around Dongguan city in China revealed continuous fallout of MP particles and fibres over the three months of autumn/winter in 2016. The study found 175–313 MP particles m^2^/day (~ 100μm or larger) of which the vast majority was fibres (>90%) [35]. Tentative recognition of weather influence is included but no examination of MP source beyond assumption of urban production.

Klein and Fischer (2019) also studied the deposition of MP in and around Hamburg, Germany [14]. This passive atmospheric deposition study was the first to compare the influence of urbanisation on atmospheric MP deposition through direct urban/rural sample comparison. The study’s findings present surprising data on the topographical effects on deposition numbers; rural (open field and forest, ~396 MP/m^2^/day) sites illustrating greater MP deposition counts (MP>50μm) than the urban areas (~215 MP/m^2^/day) [14].

Research by Bergman et al. (2019) [37] on MP content in snow illustrates MP pollution to have reached remote uninhabited locations, specifically the Arctic. Arctic samples, collected from snow deposition on ice platforms within the Fram Strait, were located ≥100km from land (Svalbard, Norway). They state that the snow contamination is atmospheric, propose that MP (>11μm, ~1760 MP/L snow) could be scavenged by snow formation and that MP are potentially arriving in these remote locations via atmospheric transport [37].

In 2016 Zhang et al. found MP in remote lake beaches on the Tibetan plateau [38]. Their research states the area is rarely visited by humans so could not explain the presence of MP. Their samples showed surface cracking and gouging of plastic particles that they felt was possibly caused by sand particles as the MP moved through the environment by wave action/saltation. The material abraded from the scratches on these <2mm MP particles could be plastic debris in the nanometer scale, <1μm [38]. Corcoran et.al. (2009) similarly found what they termed “beach weathering”, surface abrasions and cracking consistent with environmentally induced collision alongside ultra violet light degradation, to be a significant factor in the breakdown of plastics in their study of MP on Hawaiian beaches [39]. Lambert and Wagner (2016) demonstrated the breakdown of plastics to the nano scale by simulated sunlight in the laboratory yielded nanoplastic (NP) from polystyrene cup lids in just 56 days [40]. The breakdown of MP to NP by sunlight is a very real phenomena and coupled with wave action and abrasion to accelerate the process, could mean an increased number of available NP particles in the environment as larger plastic items break up over time [41–43]. In conjunction with UV, abrasion and temperature MP degradation to NP, emerging research also illustrates aquatic biota’s degradation effect. Plankton such as Antarctic Krill have been shown to cause MP degradation to NP by their digestion processes [44], *Zalerion maritimum* (a marine fungus) in laboratory conditions were found to decrease both the mass and size of MP (PE pellets 250–1000μm). There are both mechanical and biological MP to NP degradation pathways within the marine environment that may make these smaller particles available in greater quantities.

Cooper and Corcoran (2010) provide detailed descriptions of weathering effects on plastics on beaches which closely matches those described by both Zhang et al. (2016) and Cai et al. (2017). Cai et al. (2017) acknowledged the Cooper and Corcoran (2010) findings on weathering suggesting that plastics may have been transported to the sea by wind. It is perhaps just as possible that the plastics were transported by wind from a building site in the area and the abrasion is simply caused by the workers activities or vehicle traffic. This is certainly plausible for beaches or sites near population centres similar to Cooper and Corcoran’s (2010) Kauai Hawaii beach study, however less viable when presented by Zhang et al. (2016) findings on a remote Tibetan plateau.

During the period of the Cai et al. (2017) study there were several significant weather events which may have affected the material available for atmospheric fallout; three severe typhoons affected the area in October [45], cold fronts and easterly winds through November [46] as the North-easterly Monsoon sets up and the two further typhoons in December [47]_._ Severe typhoons in the China Sea entrain large amounts of sea salt and organic debris into the atmosphere and produce large wave action on the shoreline. These significant marine weather conditions suggest the need to consider possible MP atmospheric transport and source scenarios beyond microclimate urban MP generation. One possible scenario is that wave action on the shoreline together with strong winds from the typhoons entrained and carried MP particles from the beach to the city sites, ~115km inland. We know from the Allen et al. (2019) [36] study that MP particles can be transported over *at least* the medium distance which could link the China Sea atmospheric MP pollutants to the city of Dongguan. Another possibility is that the offshore weather conditions could have atmospherically entrained the plastics alongside the organic matter and SSA (that is normally found in the atmosphere post strong wind events) [48,49], producing (part of) the fallout found by Cai et.al. (2017) in Dongguan. In either of these latter scenarios it seems there is potential that the degraded particles Cai et al. (2017) found, MP that were similarly degraded to beach plastics, were similar because they may have come from the sea.

Paris is approximately 150km from the sea and in a very different climate to Dongguan. Paris does not experience Typhoons or monsoonal troughs. It does however experience a predominantly onshore wind from the North Sea/Atlantic Ocean, especially strong through winter [50]. The findings of Dris et al. (2016) showed that the winter months received generally more deposition (with the notable exception of June) [51]. The ground in winter is damp or snow covered reducing the likelihood of dust or plastic being atmospherically entrained. Potential sources for city fallout are likely to be local or regional land based for the vast majority of this recorded fallout, and the specific urban radiative microclimate hinders comparison at this early stage of investigation.

The marine air sampling campaign published by Liu et al. (2019) [16] is an illustration of long range transport and deposition of MP into the sea. Samples, taken along a transect from Shanghai to the Mariana Islands (west Pacific Ocean) at an elevation of 10m above sea level, showed MP up to 1.37MP/m^3^ (>16μm) [16]. Given the sampling locations (mid-ocean and close proximity to sea surface) there is a high likelihood of SSA and marine organic material that have been released from the sea are on these actively pumped samples. The study found more MP close to the coast, as would be expected, but also found particles at 600 nautical miles from land. It is possible that the particles were blown from land but there is also a potential for (part) of the MP found in the marine air to have come from ocean-to-atmosphere MP exchange in a similar manner to SSA.

The importance of the potential for MP or NP to act in a similar way to SSA is that the potential ocean-atmosphere exchange identifies the earths ocean surface as a possible atmospheric plastic pollution secondary source (a sink that becomes a source through bubble burst ejection and wave action). Once airborne (expelled from the ocean via bubble burst or jet ejection) MP or NP may function in a similar way to dust and salt particles [52], acting as cloud condensing nuclei (CCN) for ice or cloud nucleation [53,54]. Once airborne, dust, SSA, bacteria and sand are all known to be rained out (incorporation of particles within cloud droplets) or washed out (collision with precipitation below cloud level) of the atmosphere. If micro or nano plastic particles are similarly atmospheric it is possible they may be similarly affected (at least by the latter process) taking any adsorbed pollutants with it [55,56]. Currently the surface charge and how it could affect dust scavenging by particles is an unknown. It could be possible the triboelectric effect [52] of air passing over the plastic will statically charge the particle and make it attract dust. Van der Does et al. (2018) [57] suggest this effect could assist particles to remain aloft. The triboelectric effect may also potentially increase plastic particle’s potential to be entrained after ocean expulsion (bubble burst/jet expulsion) and transported, similar to ultragiant dust [52]. This would create larger particles and potentially change the hydrophobic nature of the particle. If MP or NP act as CCN, they may add to the total atmospheric loading of CCN, which could influence albedo and global radiation budgets [23,56]. Cloud coalescing nuclei do not have to be hygroscopic in nature, their presence is often enough to be a factor in the formation of clouds [56]. As illustrated in recent research by Ganguly and Ariya (2019) MP and NP have a high ice nucleation efficiency and may be important for cloud formation [54]. While this is theoretical and speculative, the potential for ocean-atmosphere exchange similar to SSA is a first step to the greater questions on the impact of atmospheric MP/NP.

## Field exploration of MP in sea mist–as a possible indicator of ocean MP transfer to atmosphere

A pilot investigation into sea spray and mist was conducted in field environmental conditions. The field test was designed as an indicative early look towards identifying if MP were occurring in expelled sea water droplets and if these were in the onshore blown sea air and mist.

Sea air was sampled on the French Atlantic coast from a height of 10m above MLWS (mean low water springs) for a period of 8 days. The site was on top of the first dune of Mimizan beach in the Aquitaine region, best known for its surf (44°13'10.9"N 1°17'47.8"W). The site is open to the Bay of Biscay and was ocean facing from north to south via the west, without local island or land mass interference (>1000km to the west). The study took place at the end of Autumn 2018 at a time when the number of beach goers was minimal and the meteorology is the most volatile (westerly onshore storms).

Two separate air sampler types were employed in an attempt to find the most effective system to sample sea mist and onshore airmass. Both samplers were located on the top of the dune, with the dune height noted to be 10m above sea level. The first was a standard 50L/min active air pump with a 47mm diameter circular capture area and quartz filter (Millipore QMA) placed 1.5m above ground level (height of stand was 1.5m resulting in a sample elevation of 11.5m above sea level due to the dune elevation (10m) above the sea (high tide)), providing an average pumped volume of 18m^3^/sample. The second was a Caltech Active Strand Cloudwater Collector (CASCC) [58] (cloud catcher). The CASCC2 used in this study includes a 220v fan driving air over teflon filaments which trap water droplets and direct them into a glass bottle. It was designed to capture airborne droplets in clouds/fog at 80% efficiency for >10μm droplet size (clouds/fog droplets average 10μm-30μm) [58], however it was used in this study as a possible way to capture particles suspended in sea spray fog. The CASCC2 was placed at 1.2m above ground level (height of stand for fixing,), resulting in a sample elevation of 11.2m above sea level. The CASCC2 was run at 5.8m^3^/min (an average pumped volume of 6401m^3^/sample). Corresponding sea surf water samples were collected during this period in serialised glass bottles. Both the CASCC and pumped air sampler have been used to collected atmospheric particle samples and have standardised sampling and verified efficiencies. Air particulate monitoring using the quartz filters and active air pump method have been used marine aerosol sampling [15,16] and the CASCC system has been used to collect cloud water and fog from mountain and valley locations [59–61].

Quartz filters were changed daily on the air pump and the litres of air pumped noted from the inline gas volume meter. The cloud catcher receiving bottle was also changed daily at the same time as the quartz filter. Field scientists always wore cotton clothing and stayed down wind of the equipment at all times. Blank filter material was placed in the filter cartridge in the same manner as the samples but without the pump running and removed for later analysis alongside the samples. Cloud catcher receptacle blanks were created using a sterilised bottle which was installed then removed without the fan running. Sea water was collected from the surf zone daily using a 2.5L sterilised glass bottles plunged into the surf zone at midday. The sea water sample was taken from approximately the same location each day in 0.5m deep water and the bottle was filled to capacity (2.5L). The bottle and cap were rinsed with sea water three times prior to filling to limit contamination.

Wind direction and weather conditions at the monitoring site were noted throughout the sampling periods and compared to the meteorological monitoring station at Mimizan. Mimizan local meteorology was collected from the MeteoFrance data repository [62].

All samples collected were processed following published digestions methods [36] to remove organic material from the samples. μRaman spectral analysis can be affected by biofilms or other contaminants thus it is important that material undergo a digestion process to remove unwanted organic matter [63].

Sea water samples were vacuum filtered onto Whatman 10μm cellulose filters using glass filtration equipment (triple rinsed with milliQ ultrapure water) to collect all MP and other material of 10μm or greater. Liquid collected from the cloud catcher was similarly filtered onto PTFE 0.45μm, 47mm to collect all particulates. All filtered material (from sea water, cloud collector and active air pump filters) underwent organic material removal via digestion. Filtered material was flushed into borosilicate glass vials with approximately 10ml of hydrogen peroxide (H_2_O_2_ 30% by vol). Samples were then capped with foil and heated to 55°c in a heat block for 7 days. Active air pump and cloud catcher samples were then filtered onto aluminium oxide (Anodisc) 0.2μm (25mm) filters for analysis by μRaman spectroscopy. Sea water samples underwent an additional step of density separation before final filtration, to remove all sand and sediment. Sea water samples were density separated using zinc chloride (density 1.6 Kg/L) in density separation tubes, gently agitated at 60rpm on an agitation table for 5 days. Settled material was drained off and the remaining liquid and material filtered onto Whatman 0.45μm cellulose 25mm discs for μRaman analysis (acceptable for sample of MP≥10μm diameter). The CASCC2 and air pumped samples did not present significant visible airborne dust, potentially due to the short (24 hour) sampling period, and therefore the additional density separation step was not used for these samples.

All filters were analysed with an Horiba Xplora-Plus μRaman following protocols and settings used in Allen et al. (2019) [36]. Full procedure blanks for the three sample types were run alongside the samples and their results subtracted from the described MP counts (available in the S1 Data).

## Early findings of MP in sea mist

The first two days (A1, A2) of sampling had strong onshore winds with heavy rain. The following two days (A3, A4) the wind eased substantially but remained onshore, the rain ceased however a light sea spray fog was present from the surf zone. Day 5 (A5) commenced with a light onshore sea breeze and accompanying sea mist, but wind swung round to become offshore at midday and throughout the afternoon. Days 6 through to midday day 8 (A5-A8) were influenced by offshore winds. However, late on day 8 a sea breeze convergence set up over the beach allowing for <1 m/s onshore breeze to push sea spray mist from breaking waves onto the beach (A8a). The mist was visible as a local phenomenon; it was possible to see the extent of the mist inland (~500m), offshore starting at the first line of breaking waves (glassy sea beyond) and vertically (~20-30m AMLWS). The Mimizan meteorological station recordings are collected several km inland from the site. During the sea spray mist event (A8a) the Meteo France station showed ≥4m/s offshore winds, while beach recording indicated westerly (onshore) winds of less than <1m/s. In such a sea breeze convergence the onshore sea breeze cancelled this inland offshore wind movement, resulting in a classic sea breeze convergence occurrence of onshore dense marine air movement [64]. This microclimate provided an opportunity to sample only sea spray without significant wind influence or inland airmass contribution. The local wind was too light to support plastic entrainment off the beach and therefore suggests a true sea sourced aerosol sample. While the presence of some beach sourced MPs in our samples cannot be ruled out, the results do not illustrate an increase in MP numbers with increasing onshore windspeed, therefore it is unlikely that MPs sourced from the beach are being entrained significantly in our samples in. Moreover, during the period of increase MP counts due to sea pray (A8a), the wind speed is low, thus again this indicates entrainment of beach MPs are not influencing our results.

Total for both pumped air and cloud catcher size ranges were on average 20μm (+/-13μm) and ranged between 5μm to 140μm. Particle sizes recorded from pumped filters using ImageJ software ranged between 5μm to 38μm with an average of around 13μm (+/- 4μm). This is surprising when compared to other atmospheric MP studies however this study used pumped air and not total deposition collectors which may explain the difference. It is possible the pump rate was insufficient to hold the larger and heavier particles and fibres. The largest particle captured by the cloud catcher was 140μm with a minimum of 8μm and an average 24μm (+/- 14μm) which suggests that low volume pumped filters may be underestimating particles size.

**Fig 2:**
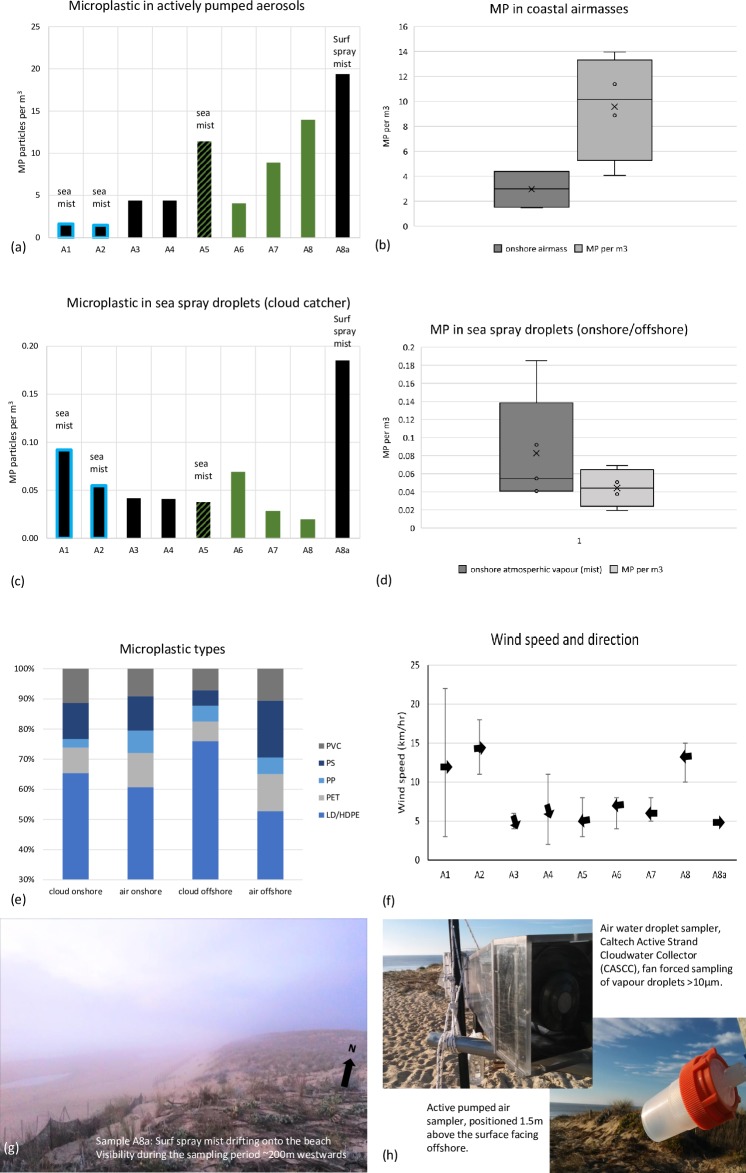
Sea mist and air mass MP counts for onshore and offshore wind and dense sea mist daily samples. Fig 2A and 2C highlights days with rain using a blue outline. Days with onshore wind are presented in black, offshore wind samples are presented in green. The final sample A8a presented very low onshore wind, with sea mist annotated on the figure. Fig 2F illustrates recorded average wind speed and direction (arrows) and the maximum and minimum wind speeds (error bars). Fig 2G illustrates the onshore (ocean) sourced atmospheric MP compared to offshore (land-based) MP counts. The sample collectors, both standardised atmospheric sampling designs, are illustrated in 2h. MP types and found represented in the samples (aerosol and droplet) are presented in 2e. MP per m^3^ refer to the respective volume of air sampled.

The CASCC2 cloud catcher MP levels in sea spray droplets during onshore wind conditions were found to be at least an order of magnitude lower (0.06±0.05, mean, 1σ, n = 9) than actively pumped total aerosols levels (7.7±6.1, mean, 1σ, n = 9), on a per m^3^ air basis. The design of the cloud sampler is to collect only material within atmospheric water droplets. As a result, the CASCC2 collected only MP that were within the water droplet fraction of the air mass. The quantities of MP found in the water droplet samples are therefore lower than the total airmass MP collected on the quartz filters. It is worth noting (as expected given CASCC2 designed function) that the CASCC2 retained more MP particles on days of high humidity (Fig 2C) and there is consequently a positive correlation between humidity and MP counts.

MP was found in all samples taken throughout the study with the sea spray mist event occurring on the last day having the highest count. Lowest air mass counts were during the heavy rain on the first two days suggesting a potential for plastics to be rained out of the atmosphere (scoured) (Fig 2A). Air water droplet showed higher MP counts during high humidity periods (rainfall periods over the first 2 days and the heavy mist periods on the last day; A1, A2, A8a, Fig 2). The water droplet trend in MP counts appeared to be inverse to the air mass counts, with greater overall air mass MP counts occurring during offshore winds, but greater water droplet MP counts occurring with onshore winds and sea mist/spray. The exception occurred with the final sample, comprised primarily of dense sea spray, A8a, where both air mass and water droplet MP counts rose notably. It is however noted that replicate samples were not collected (not possible) during this pilot study and as such the variability within a day cannot be compared to the variability between days with different weather conditions.

These early findings provide tentative early evidence to support MP exchange between ocean to atmosphere. Using the four onshore wind sample results (A1-A4) as an indicative representation of MP blown onshore, we can extrapolate to a speculative global figure of MP being blown onshore (acknowledging that the extrapolation is from a limited sample set). Taking an average size of 25μm diameter (nominal density 1g/cm^3^and volume (spherical) of 8.2 x 10^-15^m^3^/MP) particles and using the evidenced average of 2.9MP/m^3^ over a 1km stretch of beach and a marine boundary layer (MBL) of 200m in a 5m/sec wind [65], an onshore airmass gives approximately (2.96MP/m^3^) 0.024 μg/m^3^ (on a mist day the airmass contains (19.38MP/m^3^) 0.159 μg/m^3^). If 50% of world coastline (356000km) has an onshore wind, then 135,995 ton/yr of MP could be blown onshore globally (latterly terrestrially deposited or potentially blown back and deposited offshore) (calculations described in S2 Data.). Our observations were made on a moderately polluted body of water such as the Gulf of Gascoyne. With heavily polluted water bodies such as the Mediterranean Sea these figures could be much higher. This extrapolated estimation includes the assumption that MP found in the onshore wind samples were marine sourced, given the closed land mass to the west is America/Canada (>1000km). It should also be noted that SSA and other Marine aerosols reach much higher altitudes than the MBL meaning the calculated box could be much larger and hence total amounts also greater.

## Key pilot study MP findings and ocean to atmosphere exchange processes

The data from the beach air survey suggests the possibility of ocean to atmosphere transmission. Whilst there was clearly more MP coming off the land in offshore air masses, there were still significant MP numbers in onshore wind from the open Atlantic and the largest number in sea spray mist. From a review of the literature on the process of particle ejection from sea water and the global transport of SSA, organic, biologic and mineral particulates, it appears that with the presence of oceanic MP/NP there exists a potential for subsequent ocean-atmosphere transmission.

As yet, nothing is known about how plastics might behave as an aerosol but in spite of its many differences (density, shape, charge and hygroscopicity) compared to MP, Saharan dust or SSA could perhaps provide analogues for the investigation of aeolian MP transport [66]. This poses the question that with a lower density than either sand or SSA, and a greater surface area due to shape (films, fibres and irregularly shaped particles), could plastic be transported similar distances?

Whether the open ocean surface is a source of MP and NP or if the ocean acts as a line source from shoreline wave action, we suggest it is possible that plastic can be atmospherically entrained from bubble action/jet expulsion in a similar manner to SSA and other oceanic particulates. The sheer ocean surface area and length of ocean/land interface suggests that if MPs can escape the ocean and become entrained through ocean-atmospheric exchange, then this is an area that warrants future study.

## Supporting information

S1 Data Field site for the pilot study, illustrated on ESRI basemaps (used in ArcGIS) provided under the ESRI master agreement and general grant of right and restrictions basemap datasets. (DOCX)

S2 Data Exploratory extrapolation calculations of MP for 1km and 50% of the global coastline. (DOCX)

S3 Data (XLSX)

S1 Graphical abstract Exploratory extrapolation calculations of MP for 1km and 50% of the global coastline. (DOCX)
