# Supplementary material for: Examination of the ocean as a source for atmospheric microplastics
Source: PLoS One. 2020 May 12;15(5):e0232746. doi: 10.1371/journal.pone.0232746 (PMC7217454; doi:10.1371/journal.pone.0232746)
Supplement: S1 Data — (DOCX) [file pone.0232746.s001.docx]

Supplementary S1: Field site for the pilot study, illustrated on ESRI basemaps (used in ArcGIS) provided under the ESRI Master agreement and General Grant of Right and Restrictions basemap datasets

Figure S1. Map of field site location relative to local and distal land masses, illustrated on ESRI basemaps (used in ArcGIS) provided under the ESRI Master agreement and General Grant of Right and Restrictions basemap datasets


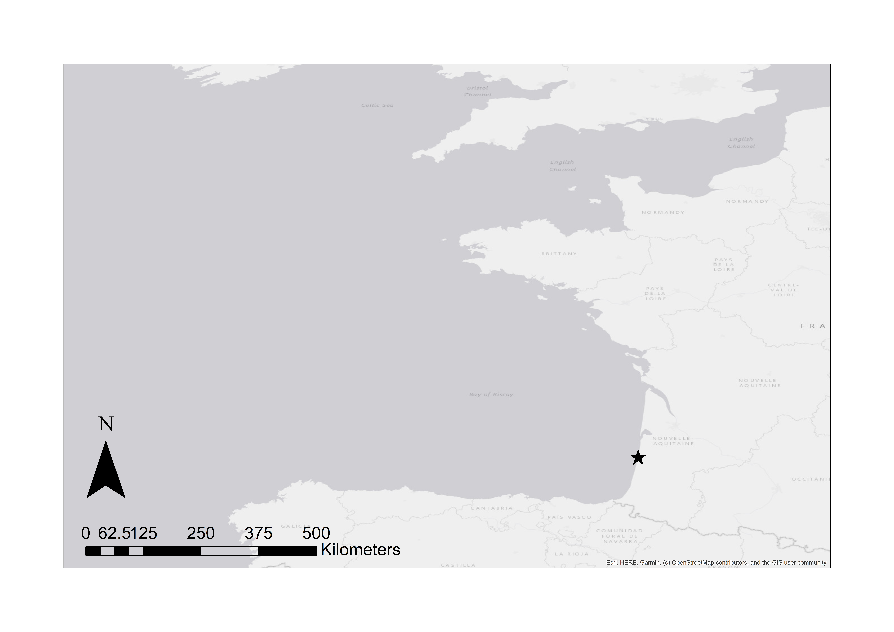

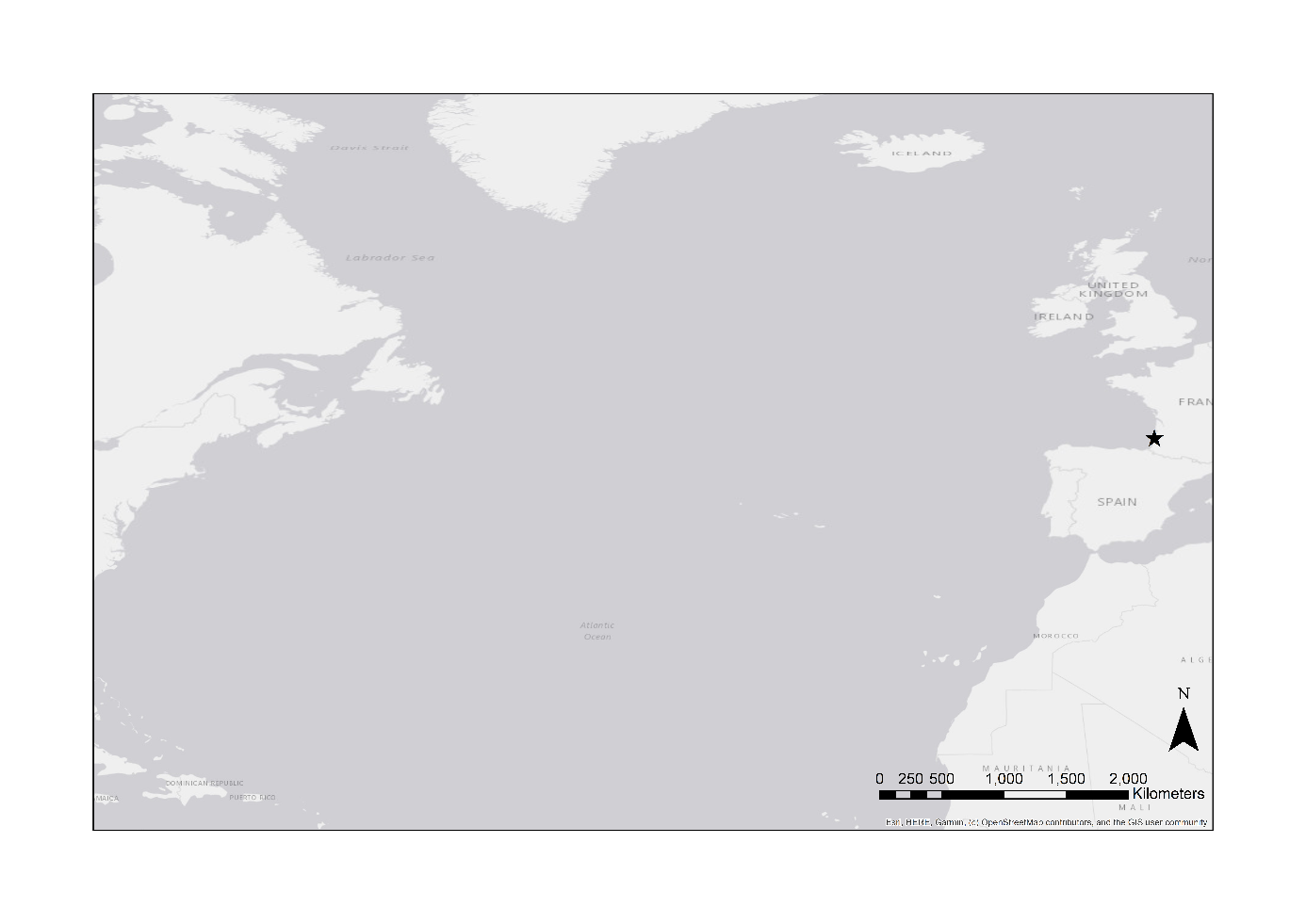


Global contextual field site map

European contextual field site map

Field monitoring location


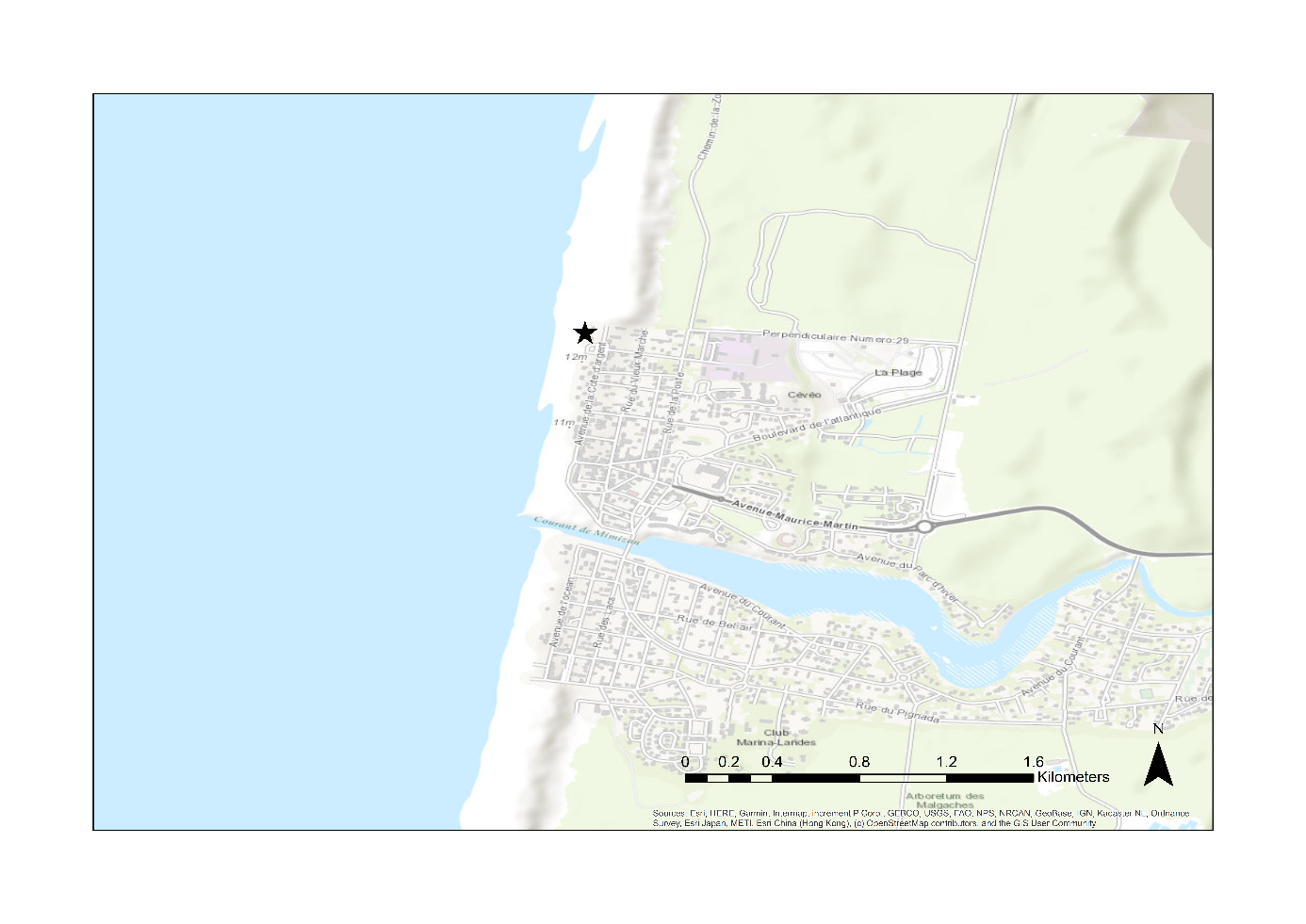


French contextual field site map

Background maps are provided by ArcGIS (Esri), with permission to reuse as specified in the Esri Master Agreements; Products and Services Terms of Use.

References for basemaps used (also noted in manuscript references):

Esri. “Global contextual field site map” [basemap]. Scale- 1:591M scale to 1:72k scale. “World Light Grey Canvas Base”. April 24, 2019. https://www.arcgis.com/home/item.html?id=87fcdf91a0f14e4a9fda40a763c6f2b8. (January 2, 2020).

Esri. “European contextual field site map” [basemap]. Scale- 1:591M scale to 1:72k scale. “World Light Grey Canvas Base”. April 24, 2019. https://www.arcgis.com/home/item.html?id=87fcdf91a0f14e4a9fda40a763c6f2b8. (January 2, 2020).

Esri. "French contextual field site map” [basemap]. Scale Not Given. "World Topographic Map". June 7, 2013. https://www.arcgis.com/home/item.html?id=6e850093c837475e8c23d905ac43b7d0. January 2, 2020).
