# Supplementary material for: Examination of the ocean as a source for atmospheric microplastics
Source: PLoS One. 2020 May 12;15(5):e0232746. doi: 10.1371/journal.pone.0232746 (PMC7217454; doi:10.1371/journal.pone.0232746)
Supplement: S2 Data — (DOCX) [file pone.0232746.s002.docx]

Supplementary S2: Exploratory extrapolation calculations of MP for 1km and 50% of the global coastline

Volume of microplastic particles (MP)

MP particle diameter = 25µm

MP particle radius = diameter/2 = 12.5µm

MP density = 1 g/cm^3^

Equation S1. $MP Volume=\frac{4}{3}\pi r^{2}$

MP mass per MP

MP mass = mass of a single MP particle in kg/MP

Equation S2. $MP mass=volume x density$

MP particles blowing onshore (#MP flux, in MP/sec)

# MP in onshore wind = 2.96 MP/m^3^ (observations for onshore wind direction samples A1-A4)

= 19.38 MP/m^3^ (observations for onshore sea mist sample A8a)

MBL(h) = Marine boundary layer (MBL) height = 200 m

Average onshore wind speed = 5 m/s (Archer and Jacobson 2005, *65*)

Length of coastline being considered = 1000m (for 1km consideration)

= 178000000 m (50% of global coastline, 356000000/2)

Air flow = Volume of air flow onshore, m^3^/s

Equation S4. $Air flow=MBL\left( h \right) x wind speed$

#MP flux = Number of MP particles blowing onshore, MP/s

Equation S5. $\#MP flux=air flow x \#MP in onshore wind$

MP mass flux onshore

Mass of MP in onshore wind in kg per second = MP mass flux (kg/s)

Equation S6. $MP mass flux=Mass \left( per MP \right) x \#MP flux$

Mass of MP in onshore wind in tonnes per year = MP mass flux (tons/yr)

Equation S7.

$$MP mass flux \left( \frac{ton}{yr} \right)=\left( MP mass flux \left( \frac{kg}{s} \right)60 x 60 x 24 x 365 \right)x 0.01$$
