# Supplementary material for: Examination of the ocean as a source for atmospheric microplastics
Source: PLoS One. 2020 May 12;15(5):e0232746. doi: 10.1371/journal.pone.0232746 (PMC7217454; doi:10.1371/journal.pone.0232746)
Supplement: S1 Graphical abstract — (DOCX) [file pone.0232746.s004.docx]

Graphical Abstract


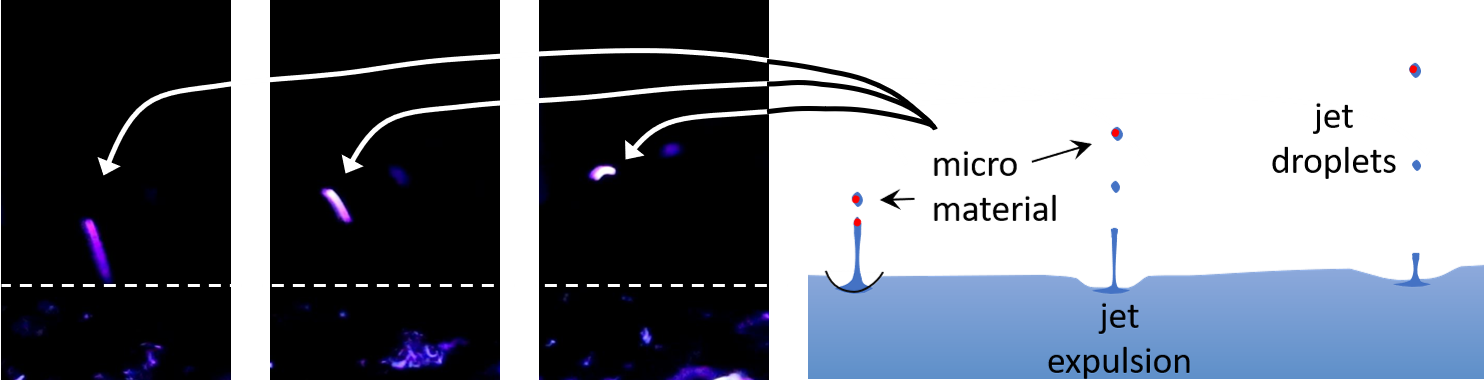


Caption: Image of a fluorescent microplastic fragment (PP, ~100µm) being ejected from a water body by jet expulsion. The fragment is illuminated using an ultraviolet light (395nm wavelength) in a darkroom. The water body (standard tap water spiked with 100µm PP fragments at approximately 200 fragments/L) is indicated below the dotted white line. Ejection bubbles were created through manual aeration of the waterbody. Images are still frames captured using a Canon EOS 800D with a 50mm lens shooting video at 50 frames per second.
